# Safety and toxicity risks of radiotherapy combined with PD-1/PD-L1 inhibitors: A comprehensive review

**DOI:** 10.1016/j.isci.2025.112882

**Published:** 2025-06-27

**Authors:** Lijing Zeng, Jing Yang, Huang Xia, Zeyuan Li, Yu Lin, Qiwei Yao, Rong Zheng

**Affiliations:** 1 Department of Radiation Oncology, Fujian Medical University Union Hospital, Fuzhou, Fujian Province, P.R. China; 2 Fujian Key Laboratory of Intelligent Imaging and Precision Radiotherapy for Tumors (Fujian Medical University), Fuzhou, Fujian Province, P.R. China; 3 Clinical Research Center for Radiology and Radiotherapy of Fujian Province (Digestive, Hematological and Breast Malignancies), Fuzhou, Fujian Province, P.R. China; 4 Department of Radiation Oncology, Fujian Cancer Hospital & Fujian Medical University Cancer Hospital, Fuzhou, Fujian 350014, P.R. China

**Keywords:** Oncology, Therapy, Immune response

## Abstract

Radiotherapy (RT) combined with PD-1/PD-L1 Inhibitors has demonstrated remarkable efficacy across various cancers. However, concerns remain regarding its safety and potential toxicity. We analyze toxicity risks of RT combined with anti–PD-1/PD-L1 therapy based on current clinical studies. While a slight increase in grade 1–2 pneumonitis has been reported in patients with lung cancer, no consistent evidence suggests a rise in ≥ grade 3 pulmonary toxicity. Additionally, the combination appears well tolerated in the central nervous system, head and neck, and hepatic malignancies. This narrative review investigates key factors that may influence the risk of toxicity in combination therapy, including the dose and fractionation of RT, sequencing with immunotherapy, timing and duration of immune consolidation, and regimen heterogeneity. This review adheres to the SANRA (Scale for the Assessment of Narrative Review Articles) guidelines.

## Graphical abstract

**Figure:**
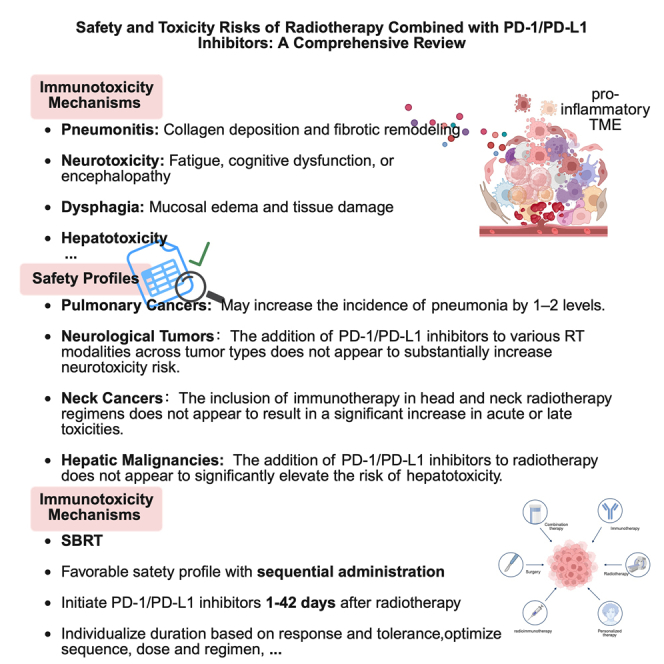

## Clinical application of radiotherapy combined with programmed cell death protein 1/programmed cell death 1 ligand inhibitors

The interaction between programmed cell death protein 1 (PD-1) and its ligand PD-L1 plays a pivotal role in tumor immune escape by inhibiting T cell activation and proliferation.^1^ Immune checkpoint inhibitors targeting the PD-1/PD-L1 axis have been developed to restore anti-tumor immune function and have demonstrated clinical benefit across multiple cancer types. However, their efficacy is frequently constrained by intrinsic or acquired resistance, limited response rates, and immune-related toxicities.^2^^,^^3^RT, long established as a cornerstone of cancer treatment, has recently been recognized for its capacity to modulate the tumor microenvironment (TME), promote immunogenic cell death, and facilitate tumor antigen presentation.^4^ These immunomodulatory effects support the combination of RT and PD-1/PD-L1 inhibitors to amplify systemic anti-tumor immunity. This combinatorial strategy has shown promising outcomes in a range of malignancies, particularly in patients with inoperable non-small cell lung cancer (NSCLC),^5^ melanoma (MM),^6^ hepatocellular carcinoma (HCC),^7^ and brain metastases (BMs). While this synergy offers substantial therapeutic potential, it also raises important safety considerations. A comprehensive understanding of toxicity profiles, risk modifiers, and underlying mechanisms is essential to guide clinical decision-making and optimize patient outcomes.

**Figure 1:**
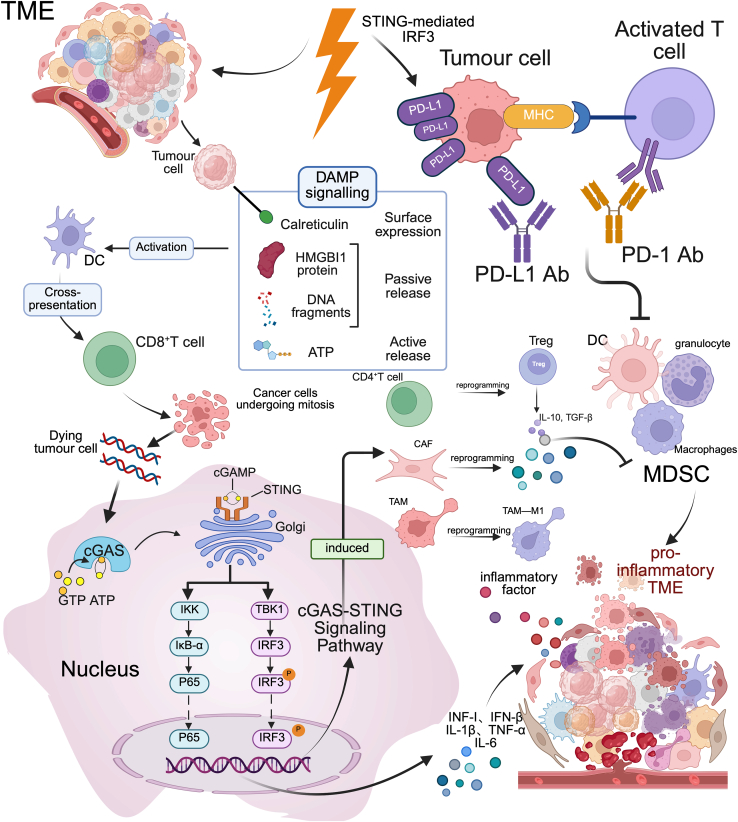
Schematic illustration of the immunological mechanisms underlying the combined effect of radiotherapy and PD-1/PD-L1 Inhibitors RT induces immunogenic cell death (ICD), resulting in the release of damage-associated molecular patterns (DAMPs) including HMGB1, calreticulin (CRT), and ATP.^18^^,^^19^^,^^20^^,^^21^^,^^22^ These signals activate dendritic cells (DCs), leading to antigen cross-presentation and priming of CD^8^⁺ cytotoxic T lymphocytes.^23^ Concurrently, RT promotes the accumulation of cytosolic double-stranded DNA (dsDNA), which activates the cyclic GMP–AMP synthase (cGAS)–(STING) pathway. cGAS senses dsDNA and generates cGAMP that activates STING, initiating its translocation to the Golgi apparatus and subsequent recruitment of TANK-binding kinase 1 (TBK1) and IκB kinase (IKK).^24^ This activation phosphorylates IRF3 and NF-κB, leading to the production of type I interferons (e.g., IFN-β) and pro-inflammatory cytokines (e.g., IL-1β, IL-6, TNF-α), thereby promoting a pro-immunogenic TME.^17^^,^^25^ cGAS–STING signaling reshapes the TME by depleting regulatory T cells (Tregs), reprogramming tumor-associated macrophages (TAMs) toward an M1-like phenotype, and suppressing the immunosuppressive activity of myeloid-derived suppressor cells (MDSCs). Cancer-associated fibroblasts (CAFs) also respond to IFN-I and inflammatory signals by secreting chemokines that facilitate immune cell recruitment. CD4^+^ T cells are polarized toward effector phenotypes that sustain adaptive immunity. However, the activation of the STING–TBK1–IRF3 axis can also upregulate PD-L1 expression on tumor cells,^8^ leading to T cell exhaustion and adaptive immune resistance. PD-1 or PD-L1 antibodies restore T cell effector function, reduce MDSC accumulation, and synergize with RT-induced immune activation, resulting in enhanced and durable anti-tumor responses. Acute activation of cGAS–STING promotes antitumor immunity, whereas prolonged signaling may induce immune dysregulation and toxicity. Chronic stimulation of cGAS–STING following RT can induce the secretion of immunosuppressive cytokines such as interleukin-10 (IL-10) and transforming growth factor β (TGF-β), which facilitate the recruitment of myeloid-derived suppressor cells (MDSCs) and Tregs 5.^26^^,^^27^ This immunosuppressive shift, paradoxically coupled with localized inflammation, may promote immune tolerance while aggravating collateral tissue damage. Within the irradiated TME, cGAS–STING activation upregulates pro-inflammatory phenotypes in CD4^+^ T cells,^28^^,^^29^ cancer-associated fibroblasts (CAFs), and macrophages.^30^^,^^31^^,^^32^ CD4^+^ T cells release cytokines such as IL-2 and IFN-γ that amplify immune cascades, while macrophages undergo M1 polarization and secrete TNF-α and IL-6^17^^,^^25^. Concurrently, CAFs also release chemokines such as CXCL12, amplifying the inflammatory response.^33^ Although this immune amplification enhances tumor clearance, it can also pose substantial risk to surrounding normal tissues. *Created in*https://BioRender.com*.*

RT and PD-1 blockade synergistically enhance systemic antitumor immunity through immune-mediated mechanisms. RT facilitates the infiltration of effector T cells, natural killer (NK) cells, and other immune populations into the TME.^8^ A pivotal mechanism involves RT-induced cytoplasmic leakage of double-stranded DNA (dsDNA) from micronuclei,^9^^,^^10^ which activates the cyclic GMP-AMP synthase (cGAS)–stimulator of interferon genes (STING) pathway.^11^ This activation promotes the production of type I interferons (IFN-I) and pro-inflammatory cytokines, supporting dendritic cell maturation and T cell activation while concurrently suppressing regulatory T cells (Tregs). Moreover, STING activation enhances nuclear factor kappa B (NF-κB) signaling, which further amplifies inflammatory responses and contributes to an immunostimulatory TME.^12^ In parallel, STING-mediated activation of interferon regulatory factor 3 (IRF3) can induce PD-L1 upregulation in tumor cells as a compensatory resistance mechanism.^13^^,^^14^ PD-1 blockade restores effector T cell function, augments TNF-α production, and disrupts tumor cell DNA repair post-RT.^15^^,^^16^^,^^17^ Together Figure 1, these immunologic cascades establish a pro-inflammatory TME that potentiates the therapeutic efficacy of PD-1/PD-L1 Inhibitors.

In the pulmonary system, CAF-derived TGF-β contributes to immune suppression and extracellular matrix remodeling, leading to excessive immune cell infiltration and collagen deposition, which are hallmarks of both radiation pneumonitis and immune-mediated pneumonitis.^34^ Similarly, in cardiac tissue, STING activation and DAMP release,^35^ and T cell infiltration^36^ contribute to myocarditis and cardiac toxicity, particularly when shared TCR antigens are present between tumor and myocardial cells 19.^37^

Tissue-specific toxicities mediated by cGAS–STING have also been observed in other organs. In the upper gastrointestinal tract, the local activation of CAFs and CD4^+^ T cells compromises mucosal integrity, contributing to dysphagia and mucositis^8^ In the central nervous system, chronic IFN-I expression can induce neurotoxicity and glial activation, potentially leading to fatigue, cognitive impairment, and other neurological symptoms.^38^ In hepatic tissue, cross-reactivity of CD8^+^ and CD4^+^ T cells against self-antigens may precipitate immune-mediated hepatitis.^39^^,^^40^

Importantly, when PD-1 blockade is administered in the setting of cGAS–STING-driven immune activation, the risk of off-target immune responses appears to be amplified. PD-1 inhibition may enhance CD4^+^ T cell-mediated immune activity^8^ not only against tumor antigens but also against self-antigens expressed in non-malignant tissues, thereby facilitating increased immune cell infiltration and robust inflammatory responses. Clinically, these processes can manifest as collagen deposition, tissue fibrosis, and a broad spectrum of systemic immune-related adverse events (irAEs).^14^

Thus, while the cGAS–STING axis plays a pivotal role in mediating anti-tumor immunity when combining RT with PD-1/PD-L1 inhibitors, it also represents a potential driver of immunotoxicity in normal tissues.

**Figure 2:**
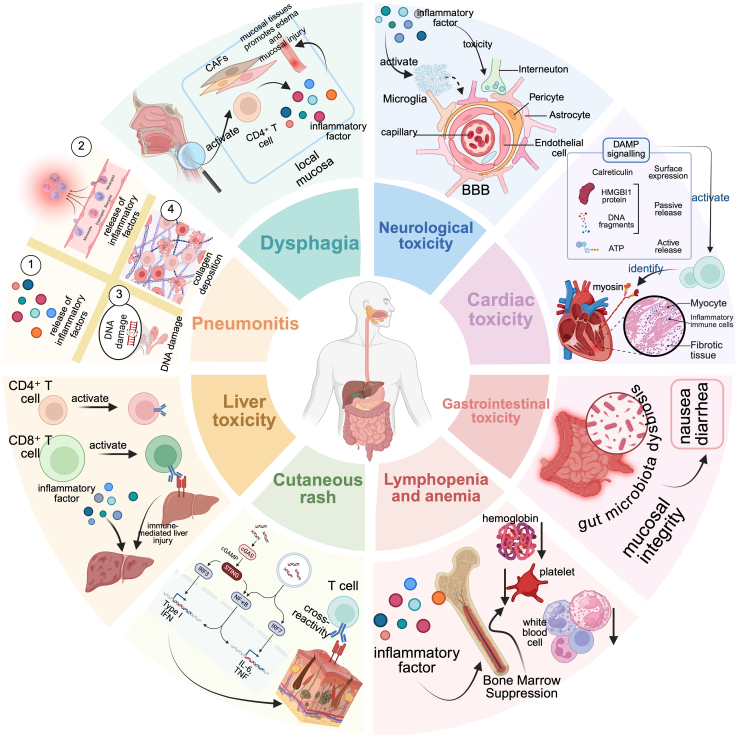
Schematic illustration of immunotoxicity mechanisms induced by combined radiotherapy and PD-1/PD-L1 inhibitors across multiple organ systems Combined radiotherapy and immune checkpoint inhibition may lead to a spectrum of immune-related adverse events involving multiple organs. **Lymphopenia and anemia:** Inflammatory cytokines and radiation-induced bone marrow suppression impair hematopoietic function, leading to a decrease in blood cells.^41^ **Dysphagia:** Activation of cancer-associated fibroblasts (CAFs) and CD4^+^ T cells in the local mucosa triggers the release of pro-inflammatory cytokines, resulting in mucosal edema and tissue damage.^8^ **Cutaneous rash:** Elevated type I interferons (IFN-I) and T cell cross-reactivity to skin-specific antigens contribute to dermatologic toxicity.^36^ **Pneumonitis:** DNA damage and inflammatory cytokines promote the infiltration of immune cells (such as T cells) into the lung parenchyma, leading to collagen deposition and fibrotic remodeling, which may progress to pneumonia.^42^ **Gastrointestinal toxicity:** Local immune activation disrupts intestinal microbial homeostasis and compromises mucosal integrity, resulting in symptoms such as nausea and diarrhea.^43^^,^^44^^,^^45^ **Cardiotoxicity:** Release of DAMPs^35^ and cytotoxic T cell responses^34^ against cardiac autoantigens, such as myosin,^46^ may trigger myocarditis, arrhythmias, and fibrosis. **Neurotoxicity:** Type I interferons and pro-inflammatory mediators crossing the blood–brain barrier (BBB) activate glial cells and contribute to neuroinflammation, potentially manifesting as fatigue, cognitive dysfunction, or encephalopathy.^38^ **Hepatotoxicity:** Activated CD8^+^ and CD4^+^ T cells recognize hepatocyte-associated antigens, leading to immune-mediated liver injury.^39^^,^^40^*Created in*https://BioRender.com*.*

Given the complexity of TME remodeling following RT and the immunological perturbations introduced by PD-1/PD-L1 inhibition, it is crucial to evaluate whether such combined regimens may exacerbate the incidence or severity of treatment-related adverse effects (AEs). As illustrated in Figure 2, these interactions underscore the need for a more comprehensive understanding of toxicity risks associated with radioimmunotherapy (IT). This review aims to address this need by synthesizing current clinical evidence on the safety profile of RT combined with PD-1/PD-L1 inhibitors, with a particular emphasis on toxicity risks when this combination is applied in the treatment of thoracic, central nervous system, head and neck, and hepatic malignancies. The reporting of this study conforms to the Scale for Assessment of Narrative Review Articles (SANRA) guidelines, a brief critical appraisal for the assessment of nonsystematic articles.

A comprehensive literature search was conducted in PubMed up to October 2024. “radiotherapy”(RT),“immunotherapy”(IT),“radioimmunotherapy”(RT/IT),“anti- Programmed cell death protein 1”(PD-1),“anti-Programmed cell death one ligand 1”(PD-L1),“Safety,” and “ adverse event” (AE) as search terms. Moreover, a manual search was also conducted by scrutinizing the reference lists of original articles, meta-analyses, and recent reviews. Inclusion criteria to identify relevant studies were:(Comparative trials involving AEs of RT + anti-PD-1/PD-L1 therapy versus radiotherapy or immunotherapy monotherapy; single-arm trials were not included. Outcome measures included: (i) data on all grades (Common Terminology Criteria for AEs [CTCAE] grades 1–5), grade 3 or 4 (CTCAE grades 3–4) AEs, treatment-related mortality (CTCAE grade 5), and treatment-related AEs leading to treatment discontinuation; and (ii) extraction and analysis focused on differences in the incidence of major AEs between monotherapy and combination therapy. The study design was a randomized clinical trial (RCT) with a parallel or crossover design. For each study, we report the first author, published year, cancer types, number of patients receiving combination therapy, number of patients receiving monotherapy, types of anti-PD (L)1, and major AEs were reported. The data are compiled in the article and systematically organized in a table format.

## Safety profiles of combined radiotherapy and programmed cell death protein 1/programmed cell death 1 ligand inhibitors

### Safety profile of combined radiotherapy and programmed cell death protein 1/programmed cell death 1 ligand inhibitors in pulmonary cancers

Thoracic radiotherapy (TRT) in combination with PD-1/PD-L1 inhibitors has been established as a standard first-line treatment for locally advanced NSCLC. Despite its therapeutic benefit, concerns persist regarding its potential to increase pulmonary toxicity. Multiple dual-arm clinical trials have investigated this issue, offering comparative data on treatment-related AEs between combination therapy and monotherapy. A synthesis of these studies reveals consistent patterns alongside some variability in findings.

The phase I KEYNOTE-001 trial,^47^ for example, reported a higher incidence of any-grade pulmonary toxicity in patients who received TRT prior to pembrolizumab (13%) than in those who did not (1%, *p* = 0.046). However, the incidence of Grade ≥3 pulmonary AEs remained comparable between groups (4% vs. 1%, *p* = 0.44). Similarly, Edwards et al.^48^ found that consolidation therapy with durvalumab following concurrent chemoradiotherapy (CRT) was associated with a significantly increased risk of Grade 2 pneumonia (hazard ratio [HR] = 1.45; *p* = 0.012), whereas the difference in Grade ≥3 pneumonia was not statistically significant (HR = 1.23; *p* = 0.20).

These results align with findings from larger trials. In the PACIFIC study^49^^,^^50^^,^^51^ Patients most commonly discontinued durvalumab due to immune-related pneumonitis and pneumonia. Importantly, the incidence of Grade 3/4 pneumonia did not differ significantly between the durvalumab and placebo groups (3.4% vs. 2.6%, *p* > 0.05). Additionally, a meta-analysis by Geng et al.,^52^ which pooled data from 20 studies involving 2,027 patients with NSCLC, demonstrated that combined therapy did not significantly increase the risk of Grade ≥3 pulmonary AEs compared with monotherapy (odds ratio [OR] = 1.24, 95% confidence interval [CI]: 0.88–1.74, *p* = 0.222), supporting the overall safety of this treatment approach.

When viewed in aggregate, as shown in Table 1, existing studies indicate that Grade 1–2 pneumonitis may be more frequently observed in patients receiving combination therapy compared to monotherapy. However, current evidence does not consistently demonstrate a significant increase in the incidence of Grade ≥3 pulmonary toxicity. These observations suggest that, in appropriately selected patients, pulmonary adverse events associated with RT and PD-1/PD-L1 inhibitors combination therapy may be clinically manageable, particularly with careful monitoring and timely supportive care. Notably, certain subgroups, including elderly patients (aged ≥70 years), may be more susceptible to severe pulmonary complications, as suggested by available subgroup analyses.^53^ This underscores the need for individualized risk assessment and treatment planning, especially in older or comorbid populations, where tolerability profiles may differ.

**Table 1:** Comparative safety outcomes of radiotherapy plus PD-1/PD-L1 inhibitors in lung cancer

(A) Study	(B) Design	(C) Histology	(D) Patients (RT + ICI)	(E) Patients (RT/ICI Alone)	(F) Target	(G) RT Dose (Gy)	(H) Follow-up	(I) Adverse Events (AE)
Narek et al.^47^	Retrospective	NSCLC	24 (TRT+anti-PD-1)	73 (anti-PD-1)	PD-1	NR	32.5 months (range 29.8–34.1)	TRPT (Any grade: 13% vs. 1%, *p* = 0.046; ≥G3: 4% vs. 1%, *p* = 0.44)
Scott et al.^51^	Prospective	NSCLC	473 (CRT+ anti-PD-1)	236 (CRT+placebo)	PD-1	54–66	14.5 months (range 0.2–29.9)	G3–4 AEs: 29.9% vs. 26.1%; Pneumonitis G3–4: 4.4% vs. 3.8%
Fiorica et al.^54^	Retrospective	NSCLC	15	20 (anti-PD-1)	PD-1	8–36	7.3months	G3 irAE: 1 in each group; G2 skin AE (PD-1: 2, RT + PD-1: 1)
Gishan et al.^55^	Retrospective	NSCLC	65	20 (anti-PD-1)	PD-1	RT + ICI: 30 (8–66); Con: 20 (6–50)	15months	Any AE: 18% vs. 25% (*p* = 0.79); G3–4: 8.7% vs. 12.5% (*p* = 0.677)
Yamaguchi et al.^56^	Retrospective	NSCLC	66	58 (anti-PD-1)	PD-1	CTR: 50–60; palliative: 30–40; bone: 8–30; cranial: 30–50	NR	TRPT: 20% vs. 14%
Willemijn et al.^57^	Retrospective	NSCLC	36	40 (anti-PD-1)	PD-1	NR	NR	Fatigue: 51% vs. 37% (*p* = 0.05); Pneumonitis: 26% vs. 8% (*p* = 0.06)
James et al.^58^	Prospective	mNSCLC	60	40 (anti-PD-1)	PD-1	50/4 fx; 45/15 fx	20.4 months (range 1.4–30.2)	G3 AEs: 23 (RT + PD-1)
Suchita et al.^59^	Prospective	NSCLC	9 (RT+ anti-PD-1/PD-L1)	9 (anti-PD-[L]1	PD-1/PD-L1	9	NR	G1 fatigue: 44% vs. 66%
Evangeline et al.^60^	Retrospective	NSCLC	102	167 (anti-PD-1)	PD-1	NR	NR	Any grade: 21% vs. 20%; ≥G3: 13% vs. 8%
Wang et al.^61^	Retrospective	NSCLC	59	93 (anti-PD-1)	PD-1	NR	8.1months (range 1.3–29.9)	AE (all grades): 97%; G1–2: 81%; G3–4: 15%
Shinobu et al.^62^	Retrospective	NSCLC	139	392 (anti-PD-[L]1)	PD-(L)1	NR	NR	irAE: 35.9% vs. 31.9% (*p* = 0.402); Pneumonitis: 9.4% vs. 7.0% (*p* = 0.467)
Go Saito et al.^63^	Retrospective	NSCLC	225	77 (CRT)	PD-1	Median 60 (14–70.4)	8.4 months (range 1.5–15.7)	Pneumonitis (All grade: 85% vs. 78%; ≥G3: 7% vs. 8%; G5: 1% vs. 3%)
Nasser K Altorkiet al.^64^	Prospective	NSCLC	30	30 (anti-PD-1)	PD-1	8 × 3 fx	NR	G3–4 AE: 20% vs. 17%
Zhou et al.^65^	Prospective	NSCLC	255	126 (CRT)	PD-L1	NR	NR	SAE: sugemalimab 15%, placebo 10%
Edwards et al.^48^	Retrospective	NSCLC	1005	989 (CRT)	PD-L1	NR	1.9 years (durvalumab); 5.1years (no durvalumab)	Pneumonitis: G2 (HR = 1.45; 95% CI: 1.09–1.93; *p* = 0.012); G3–5 (*p* = 0.2)

AE = adverse event; CRT = chemoradiotherapy; CT = conventional thoracic RT; fx = fractions; G = grade; ICI = immune checkpoint inhibitor; irAE = immune-related adverse event; NSCLC = non-small cell lung cancer; mNSCLC = metastatic NSCLC; NR = not reported; PD-1 = programmed death-1; PD-L1 = programmed death-ligand 1; RT = radiotherapy; SAE = serious adverse event; TRPT = treatment-related pulmonary toxicity.

Note: All comparative data in the tables (indicated as “vs.”) represent outcomes in the *experimental group* (RT + PD-1/PD-L1 inhibitors) versus the *control group* (radiotherapy or immunotherapy alone).

Beyond treatment-specific factors, patient characteristics, particularly age and comorbidities, may significantly influence pulmonary toxicity risk in the context of TRT combined with immune checkpoint blockade. Although pivotal trials such as PACIFIC^49^^,^^50^^,^^51^ and PEMBRO-RT^57^ did not exclude elderly individuals or patients with underlying conditions, subgroup analyses stratified by these variables are often lacking. Age-related immune senescence may exacerbate inflammatory responses to both radiotherapy and immunotherapy, thereby increasing susceptibility to pneumonitis. In addition, patients with chronic pulmonary diseases such as chronic obstructive pulmonary disease (COPD) may exhibit baseline inflammation that predisposes them to more severe immune-mediated pulmonary injury. Nevertheless, clinical data addressing these interactions remain limited. Future prospective trials should include more heterogeneous patient populations and incorporate stratified toxicity analyses to inform precision treatment and optimize safety in a real-world setting.

### Safety profile of combined radiotherapy and programmed cell death protein 1/programmed cell death 1 ligand inhibitors in neurological tumors

BM are a frequent and clinically challenging complication in advanced malignancies such as NSCLC, MM, and breast cancer (BC), often associated with poor prognosis.^66^ Standard therapeutic approaches include surgery, whole-brain radiotherapy (WBRT), stereotactic radiosurgery (SRS), or their combinations.^67^ Recently, the incorporation of PD-1/PD-L1 inhibitors into brain-directed radiotherapy has emerged as a promising strategy. As such, evaluating the neurological safety of this combined modality is of critical clinical importance.

Several comparative studies have explored the potential impact of adding PD-1/PD-L1 inhibitors to brain RT on neurological toxicity. While findings vary across study designs and patient populations, many reports have not identified a clear increase in the incidence of severe (Grade ≥3) neurologic AEs. These results may suggest that, in selected settings, the neurological safety profile of this combination appears to be generally acceptable.

For instance, Hubbeling et al.^68^ assessed 163 patients with NSCLC with BM and found that the incidence of both any-grade and Grade ≥3 AEs was comparable between the combination therapy group ([ICI]+, *n* = 50) and the RT-alone group (ICI–, *n* = 113), with no statistically significant differences. Shepard et al.^69^ echoed these findings, reporting similar rates of radiation necrosis (RN), intratumoral hemorrhage, and peritumoral edema between patients receiving SRS combined with PD-1/PD-L1 inhibitors and those receiving SRS alone.

This pattern is further supported by a multicenter study conducted by AIRO,^70^ which evaluated 150 patients with NSCLC-BM. The incidence of any-grade RN (20% vs. 22%, *p* = 0.83) and Grade ≥2 RN (5% vs. 4%, *p* = 1.00) did not differ significantly between the SRT + PD-1/PD-L1 group and the SRT-alone group. Similarly, a propensity score-matched analysis of 115 patients with NSCLC-BM^71^ demonstrated comparable rates of Grade 3 AEs in the combination group versus the monotherapy group (3% vs. 1%, *p* = 0.15). Further supporting the neurological tolerability of combined therapy, Li et al.^72^ analyzed 210 patients with NSCLC-BM treated with CRT. Among those who received PD-1/PD-L1 inhibitors within six months (*n* = 56) and those who did not (*n* = 154), the incidence of white matter lesions (WMLs) and radiation necrosis was similar, indicating no additional neurological risk attributable to immunotherapy. Beyond NSCLC, Ma et al.^73^ examined the neurological safety of PD-L1 inhibitor-based combination therapy in 46 patients with extensive-stage small cell lung cancer (ES-SCLC). These results mirrored NSCLC cohorts, with similar rates of brain injury (55.65% vs. 54.54%), and Grade 3 AEs (22.22% vs. 27.27%) were comparable between the combination and monotherapy arms.

Taken together Table 2, these data suggest that adding PD-1/PD-L1 inhibitors to various RT modalities across tumor types does not substantially increase neurotoxicity risk. Although minor differences exist between studies, the overall trend supports the neurological safety of this treatment approach, particularly when applied with appropriate patient selection, treatment planning, and surveillance.

**Table 2:** Comparative safety outcomes of radiotherapy plus PD-1/PD-L1 inhibitors in central nervous system tumors

(A) Study	(B) Design	(C) Histology	(D) Patients (RT + ICI)	(E) Patients (RT/ICI Alone)	(F) Target	(G) RT Dose (Gy)	(H) Follow-up	(I) Adverse Events (AE)
Harper et al.^68^	Retrospective	NSCLC-BM	50	113 (RT)	PD-1	Median 1 (1–11)	16 months (range 1–140)	AE (all grade): 50%–90% vs. 49%–97%; G3-4:9%–13% vs. 5%–8%; Sympomatic: 13%–31% vs. 5%–34%
Charu et al.^74^	Retrospective	NSCLC-BM	39	46 (CRT)	PD-1	NR	NR	RNC: 10.2% vs. 10% (*p* = 0.7)
Matthew et al.^69^	Retrospective	NSCLC-BM	17	34 (anti-RT)	PD-1/PD-L1	SRS+ICI: 18.4 ± 2.3; ICI: 19.3 ± 2.5	NR	RN and IH 5.9% vs. 2.9% (*p* = 0.99)
Willemijn et al.^75^	Retrospective	NSCLC-BM	72	76 (anti-PD-1)	PD-1	NR	33 months	No new safety concerns were noted.
Silvia et al.^70^	Retrospective	NSCLC-BM	100	50 (SRS)	PD-(L)1	Median 28.9 (18–33)	RT: 19 months; RT + ICI: 23 months	Any grade RN: 22% vs. 20% (*p* = 0.83); ≥G2 RN: 4% vs. 5% (*p* = 1.0)
Mohammed et al.^71^	Retrospective	NSCLC-BM	80	235 (SRS)	PD-(L)1	Median 51.0 (47.5–60.0)	NR	G3 AE: 3% vs. 1% (*p* = 0.15)
Ma et al.^73^	Retrospective	SCLC-BM	15	31 (BRT)	PD-L1	NR	NR	RIL: 55.65% vs. 54.54%
Li et al.^72^	Retrospective	NSCLC-BM	56	154 (CRT)	PD-(L)1	Median 27.4 (1.0–50.4)	NR	All grade RN: 14.3% vs. 5.8% (*p* = 0.090); WMLs: 62.5% vs. 69.5%–13% (*p* = 0.339)
Lu et al.^76^	Retrospective	NSCLC-BM	74	39 (anti-PD-[L]1	PD-1/PD-L1	WBRT: 30 Gy (10 fx) or 40 Gy (20 fx)	NR	RT + ICI may increase risk of radiation therapy–related AEs
Guo et al.^77^	Prospective	NSCLC-BM	280	28 (anti- PD-L1)	PD-L1	NR	NR	NAE: 65% vs. 43%; G3/4: 3% vs. 0%; SNAE: 8% vs. 4%

NSCLC-BM = Non-small cell lung cancer with brain metastases; SCLC-BM = Small cell lung cancer with brain metastases; SRS = Stereotactic radiosurgery; WBRT = Whole brain radiotherapy; BRT = Brain radiotherapy; RN = Radiation necrosis; RIL = Radiation-induced leukoencephalopathy; WMLs = White matter lesions; NAE = Neurological adverse events; SNAE = Serious neurological adverse events; niBM = Non-irradiated brain metastases group; iBM = Irradiated brain metastases group.

### Safety profile of combined radiotherapy and programmed cell death protein 1/programmed cell death 1 ligand inhibitors in head and neck cancers

Head and neck cancers (HNCs) are common malignancies, with 70–80% of head and neck squamous cell carcinoma (HNSCC)cases diagnosed at a locally advanced stage (III/IV).^78^ Nasopharyngeal carcinoma (NPC) is highly prevalent in China and Southeast Asia, accounting for nearly half of global cases.^79^ While cisplatin-based chemoradiotherapy remains the standard for HNSCC and intensity-modulated radiotherapy (IMRT) improves local control in NPC, distant metastasis and recurrence remain major challenges. The addition of PD-1/PD-L1 Inhibitors to radiotherapy offers a new treatment paradigm, improving survival in both locally advanced and recurrent/metastatic settings, though safety concerns warrant careful evaluation.

In recent years, PD-1/PD-L1 Inhibitors have been investigated as potential additions or alternatives to existing RT-based regimens. In a large-scale randomized controlled trial involving 425 patients with recurrent or metastatic nasopharyngeal carcinoma, sintilimab was added to standard CRT to evaluate its safety profile.^80^ Patients were randomized in a 1:1 ratio to receive sintilimab (*n* = 210) or standard therapy (*n* = 215), stratified by treatment center and disease stage (III vs. IV). All patients underwent induction chemotherapy with gemcitabine and cisplatin, followed by cisplatin-based CRT. After a median follow-up of 41.9 months (IQR: 38.0–44.8), the incidence of Grade 3–4 AEs was similar across both groups: mucositis (33% vs. 30%), leukopenia (26% vs. 22%), neutropenia (24% vs. 21%), anemia (16% vs. 11%), thrombocytopenia (16% vs. 12%), nausea (14% vs. 15%), and vomiting (11% in both groups). Late-onset all-grade AEs were also comparable, with dry mouth (73% vs. 70%), dysphagia (33% vs. 32%), and impaired hearing (35% vs. 34%) occurring at similar rates.

Additional safety data come from randomized studies directly comparing PD-1/PD-L1 inhibitors with cetuximab-based RT. A phase III trial in LA-HNSCC^81^ randomized 133 patients to receive either pembrolizumab + RT (*n* = 67) or cetuximab + RT (*n* = 66), with median follow-up durations of 25.6 and 25.8 months, respectively. Grade 5 AEs occurred in both arms (5 cases in the cetuximab-RT group and 4 in the pembrolizumab-RT group), attributed to events such as sepsis, myocardial infarction, arterial thrombosis, and unexplained death. The most common Grade ≥3 AEs in both groups included mucositis, radiation-induced dysphagia, dermatitis, and rash. Notably, the cetuximab-RT group had slightly higher frequencies of these events, except for dysphagia.

The REACH trial further evaluated the safety of a novel triple combination of avelumab,^82^ cetuximab, and IMRT in both cisplatin-eligible and -ineligible patients. In total, 41 patients in experimental arms (groups B and C) received this regimen. The most frequently reported all-grade AEs were mucositis (98%), radiodermatitis (95%), dysphagia (83%), and rash (78%). The most frequent Grade ≥3 AEs were mucositis (*n* = 21), radiodermatitis (*n* = 20), and dysphagia (*n* = 16). Other Grade ≥3 toxicities occurring in >5% of patients included lymphopenia (*n* = 6), catheter-related infections (*n* = 3), and oral pain (*n* = 3). Notably, the rate of Grade ≥4 acute AEs was 12% (5/41), aligning closely with historical controls from GORTEC trials (CRT: 18%, cetuximab-RT: 15%).

In summary, Table 3, across various regimens and PD-1/PD-L1 inhibitors, the addition of immunotherapy to head and neck RT protocols has not led to a marked increase in either acute or late toxicities. Mucositis, dermatitis, and dysphagia remain the most frequently observed and clinically relevant adverse events, yet their incidence remains comparable to that reported with standard regimens. These data suggest that combining immunotherapy with RT in HNSCC and NPC is generally safe and tolerable; however, continued post-treatment surveillance and longer-term follow-up are essential to confirm these findings and detect any late-emerging complications.

**Table 3:** Comparative safety outcomes of radiotherapy plus PD-1/PD-L1 inhibitors in head and neck cancer

(A) Study	(B) Design	(C) Histology	(D) Patients (RT + ICI)	(E) Patients (RT/ICI Alone)	(F) Target	(G) RT Dose (Gy)	(H) Follow-up	(I) Adverse Events (AE)
Tao et al.^80^	Prospective	SCCHN	41 (RT + avelumab + cetuximab)	21 CRT	PD-L1	High-risk CTV: 69.96; Low-risk CTV: 52.8	NR	Grade ≥3 AE: mucositis (51%), radiodermatitis (49%), dysphagia (39%)
Tao et al.^81^	Prospective	SCCHN	67 (RT + anti-PD-1)	66 (RT + cetuximab)	PD-1	2 High-risk CTV: 69.96; Low-risk CTV: 52.8	25 months	Grade ≥3 AE: mucositis, dysphagia, radiodermatitis, rash. All-grade radiation dysphagia: 78% vs. 65%
Liu et al.^82^	Retrospective	NPC	210 (CRT-*anti*-PD-1)	215 (CRT)	PD-1	69.96	1.9 months (range 38.0–44.8)	Grade ≥3 AE: mucositis (33% vs. 30%), leukopenia (26% vs. 22%), neutropenia (24% vs. 21%), anemia (16% vs. 11%), thrombocytopenia (16% vs. 12%)

NPC = Nasopharyngeal carcinoma; SCCHN = Squamous cell carcinoma of head and neck (SCCHN); CTV = Clinical Target Volum.

### Safety profile of combined radiotherapy and programmed cell death protein 1/programmed cell death 1 ligand inhibitors in hepatic malignancies

Liver cancer remains the third leading cause of cancer-related mortality worldwide, with HCC accounting for over 80% of cases.^83^ As the clinical application of RT combined with PD-1/PD-L1 inhibitors continues to expand in HCC treatment, it is critical to assess the hepatic toxicity profile of this therapeutic strategy. While high-quality comparative data are currently limited, preliminary evidence from retrospective and early-phase studies indicates that the addition of RT to PD-1/PD-L1 blockade may not substantially elevate the risk of liver-related toxicity.

In a representative retrospective study, Su et al.^84^ compared 197 patients with HCC who received either triple therapy (PD-1 inhibitors + anti-angiogenic therapy + intensity-modulated radiation therapy [IMRT], *n* = 54) or dual therapy (PD-1 inhibitors + anti-angiogenic therapy, *n* = 143). Patients were treated with PD-1 inhibitors every three weeks and daily anti-angiogenic agents until disease progression or unacceptable toxicity. The overall incidence of any-grade adverse events was comparable between groups (*p* > 0.05). Notably, no statistically significant differences were observed in key hepatic or hematologic toxicities, including aspartate aminotransferase (AST) elevation (Grade 1–2: 16.7% vs. 9.8%; ≥ Grade 3: 3.7% vs. 2.1%, *p* = 0.314), leukopenia (Grade 1–2: 53.7% vs. 40.6%; ≥ Grade 3: 7.4% vs. 5.6%, *p* = 0.173), and thrombocytopenia (Grade 1–2: 44.4% vs. 36.4%; ≥ Grade 3: 5.6% vs. 4.9%, *p* = 0.541). These findings suggest that, within the context of this study, the addition of RT to immunotherapy and anti-angiogenic therapy did not appear to significantly exacerbate hepatic or hematologic toxicities. However, further prospective validation is warranted.

Further evidence is provided by Zhang et al.,^85^ who conducted a propensity score-matched analysis comparing 30 patients treated with RT plus anti-PD-1 therapy and 30 patients receiving RT alone. Most liver-related toxicities were comparable between the two groups, including ALT elevation (≥ Grade 1: 43.4% vs. 23.3%, *p* = 0.171), albumin decrease (≥ Grade 1: 63.3% vs. 36.7%, *p* = 0.071), and Grade 2 albumin reduction (30.0% vs. 16.7%, *p* = 0.360). A higher incidence of AST elevation (≥ Grade 1: 66.7% vs. 40.0%, *p* = 0.020) was observed in the combination group, which may warrant attention. However, this laboratory abnormality did not translate into clinical liver failure or severe hepatotoxicity: the incidence of radiation-induced liver disease (RILD) was identical in both groups (23.3%, *p* = 1.000), and no Grade 4/5 hepatic AEs were reported. Furthermore, there were no instances of Grade 3 toxicities related to ALP, bilirubin, or albumin, supporting an overall favorable hepatic safety profile.

Several single-arm studies further support these findings,^86^^,^^87^^,^^88^^,^^89^ which also demonstrated a low incidence of liver toxicity in patients receiving RT combined with PD-1/PD-L1 inhibitors. However, due to the absence of control arms, the generalizability of these results is limited.

Taken together, and as shown in Table 4, current comparative data indicate that the addition of PD-1/PD-L1 inhibitors to radiotherapy does not appear to significantly elevate the risk of hepatotoxicity in patients with HCC. Most reported hepatic AEs are mild to moderate in severity and are generally manageable with standard clinical interventions. However, sporadic findings, including increases in AST levels, highlight the importance of ongoing monitoring during treatment. Considering the limited number of robust, large-scale comparative studies, further prospective investigations involving diverse patient populations are needed to more definitively characterize the hepatic safety profile and long-term tolerability of this combined therapeutic strategy.

**Table 4:** Comparative safety outcomes of radiotherapy plus PD-1/PD-L1 inhibitors in hepatocellular carcinoma

(A) Study	(B) Design	(C) Histology	(D) Patients (RT + ICI)	(E) Patients (RT/ICI Alone)	(F) Target	(G) RT Dose (Gy)	(H) Follow-up	(I) Adverse Events (AE)
Su et al.^84^	Retrospective	HCC	54 (anti-PD-1+IMRT+AAT)	143 (anti-PD-1+AAT)	PD-1	NR	NR	no statistically significant difference in the incidence of AE between the two groups (*p* < 0.05)
Zhang et al.^85^	Retrospective	HCC	30	66 (RT)	PD-1	Median 51.0 (range: 47.5–60.0)	NR	AST ≥ Grade 1: (66.7%vs.40.0%,*p* = 0.020); Radiation-induced liver disease: (23.3%vs.23.3%,*p* = 1.000)

HCC = Hepatocellular Carcinoma; IMRT = Intensity-Modulated Radiotherapy; AAT = Anti-Angiogenic Therapy.

## Influencing factors

### Impact of radiotherapy fractionation on toxicity in combination with programmed cell death protein 1/programmed cell death 1 ligand inhibitors therapy

The fractionation regimen of RT plays a critical role in the development of toxicity profiles during combination therapy with PD-1/PD-L1 inhibitors. Conventional fractionated radiotherapy (CFRT) typically involves delivering 2 Gy doses over several weeks to large tumors (>5–10 cm^3^), while stereotactic body radiotherapy (SBRT) uses fractionated high-dose radiation (>5 Gy per fraction) combined with submillimeter-level precision and smaller target volumes.^90^ These fundamental differences not only impact tumor control and immune activation but also influence the spectrum and severity of treatment-related toxicities in RT combined with PD-1/PD-L1 inhibitors regimens.

Several clinical studies directly compared toxicity outcomes between CFRT and SBRT in the context of combined immunotherapy.^58^ In a phase I/II trial involving patients with metastatic non-small cell lung cancer, patients received pembrolizumab combined with SBRT (50 Gy in 4 fractions) or CFRT (45 Gy in 15 fractions). The SBRT group had a lower incidence of grade 3 or higher adverse events, with only 2 cases reported (right ventricular dysfunction and pneumonia), while the CFRT group reported 5 cases, including pneumonia, rash, and pleural effusion. Additionally, SBRT was significantly associated with a lower incidence of lymphopenia (19.0% vs. 47.0%, *p* = 0.003), a key factor affecting immune function during immunotherapy.

In another multicenter randomized trial, the efficacy of durvalumab–tremelimumab in combination with or without RT was evaluated for the treatment of metastatic NSCLC. Patients receiving high-fractionated radiotherapy (HFRT; 8 Gy every other day) had a lower incidence of grade 3 or higher adverse events (12%) compared to those receiving low-dose fractionated radiotherapy (LDFRT; 2 Gy × 4) or no radiotherapy (15%). Notably, the LDFRT group reported one grade 5 fatal adverse event (respiratory failure), further supporting the safety advantage of the HFRT regimen.^91^

In conclusion, emerging clinical evidence suggests that SBRT and other hypofractionated regimens may be associated with lower or at least comparable rates of high-grade toxicities compared to CFRT when combined with PD-1/PD-L1 inhibitors. From a mechanistic standpoint, SBRT appears to better preserve circulating lymphocytes, mitigate radiation-induced hypoxia, and enhance immunogenic cell death through caspase-3 activation and type I interferon signaling.^92^^,^^93^^,^^94^^,^^95^ In contrast, CFRT may more profoundly disrupt tumor vasculature, exacerbate hypoxic conditions, and potentially compromise effective immune priming.^96^ These mechanistic differences may underlie the observed variability in both efficacy and toxicity across fractionation strategies. From a clinical perspective, elucidating the influence of RT fractionation on immunotherapy-related toxicity is essential for optimizing treatment protocols. While early-phase studies offer promising insights, prospective trials with larger, stratified cohorts are needed to define the most effective and safest dose-fractionation schedules across diverse tumor types. Ultimately, individualized radiotherapy planning that incorporates tumor location, immunologic profile, and patient-specific factors may help to enhance therapeutic synergy while minimizing AEs.

### Influence of radiotherapy and programmed cell death protein 1/programmed cell death 1 ligand inhibitors sequencing on treatment-related toxicity

Following the discussion on dose and fractionation, the timing of immunotherapy administration relative to radiotherapy has also emerged as a key determinant of treatment-related toxicity. Whether delivered sequentially or concurrently, the interaction between RT and PD-1/PD-L1 inhibitors may significantly influence immune activation and adverse event profiles. While current evidence remains heterogeneous, several studies suggest that initiating PD-1/PD-L1 inhibitor therapy after RT may be associated with a more favorable safety profile in certain clinical contexts.

For example, in the PACIFIC trial,^49^^,^^50^^,^^51^ durvalumab consolidation following CRT became the standard of care for unresectable stage III NSCLC, demonstrating durable survival benefits and a manageable toxicity profile. Similarly, in the KEYNOTE-001^47^ and PEMBRO-RT^57^ trials, pembrolizumab was administered sequentially after RT and was associated with low rates of high-grade AEs. These findings collectively suggest that delaying the initiation of PD-1/PD-L1 inhibitors following RT may help mitigate early immune-related toxicities.

Conversely, the concurrent administration of RT and PD-1/PD-L1 INHIBITORS has yielded mixed safety outcomes. The PACIFIC-2 phase III trial evaluated concurrent durvalumab during CRT,^97^ followed by durvalumab maintenance. Although overall pneumonitis rates were similar between the concurrent and sequential arms (28.8% vs. 28.7%), the concurrent group experienced a notably higher rate of early treatment discontinuation due to AEs within the first four months (14.2% vs. 5.6%). Early immune-related toxicities, including pulmonary AEs and infections, were implicated in treatment interruptions and, in some cases, may have contributed to treatment-related mortality.

Differences in trial design may partially explain the observed discrepancies between PACIFIC and PACIFIC-2. In PACIFIC-2, patients were randomized prior to CRT, which included individuals who subsequently developed progressive disease or severe toxicity during treatment. In contrast, PACIFIC enrolled only patients who had completed CRT without progression or unacceptable toxicity, effectively selecting for a more treatment-tolerant population. These nuances underscore the importance of contextualizing outcomes within the framework of trial eligibility and highlight the influence of patient selection on safety and efficacy findings.

Retrospective analyses further support the safety of delaying PD-1/PD-L1 inhibitor therapy. In one study,^98^ postponing systemic therapy until after disease progression was associated with a significantly reduced risk of Grade ≥2 toxicity (odds ratio = 0.35; 95% confidence interval [CI]: 0.15–0.70; *p* < 0.001). The cumulative AE incidence was 13% in the delayed group, compared to 24% in those receiving early treatment. Similarly, in patients with melanoma brain metastases treated with SRS,^99^ lower rates of treatment-associated brain necrosis were observed when PD-1/PD-L1 inhibitors were administered post-SRS. For example, brain necrosis incidence was 7.4% in those receiving pembrolizumab monotherapy after SRS, and hazard analysis indicated a trend toward reduced risk with delayed PD-1/PD-L1 INHIBITORS administration (hazard ratio = 0.55; 95% CI: 0.28–1.08; *p* = 0.081).

From a mechanistic standpoint, concurrent RT and PD-1/PD-L1 INHIBITORS administration may exacerbate inflammatory processes in irradiated tissues, increasing the likelihood of immune-related AEs. Sequential administration, by contrast, may allow for the partial resolution of radiation-induced inflammation prior to ICI exposure, potentially reducing toxicity risk. However, cross-study comparisons remain limited by variability in the operational definitions of “concurrent” and “sequential” treatment. Reported time intervals for “concurrent” ICI administration range from within 1 week^100^ to as long as 5.5 months,^101^ with commonly used windows including 14 days^102^^,^^103^^,^^104^ to 1 month.^105^^,^^106^^,^^107^ Such heterogeneity hampers interpretation and underscores the need for standardized definitions in future investigations.

Taken together, current data suggest that the sequential administration of PD-1/PD-L1 inhibitors following radiotherapy may be associated with a more favorable toxicity profile in certain clinical settings. However, this observation should be interpreted with caution. Differences in the incidence of immune-related adverse events, such as those reported in the PACIFIC and PACIFIC-2 studies, may not only be due to differences in treatment sequences but also reflect differences in trial design, inclusion criteria, baseline patient characteristics, and disease burden. Therefore, it is inappropriate to attribute observed safety differences exclusively to the timing of PD-1/PD-L1 inhibitors administration. The interplay between treatment sequence and other clinical variables must be carefully accounted for. Prospective, stratified trials with standardized definitions of concurrent and sequential therapy are essential to more precisely determine the optimal sequencing strategy for minimizing toxicity while maintaining efficacy.

### Timing and duration of programmed cell death protein 1/programmed cell death 1 ligand therapy in relation to toxicity risk

The timing and duration of PD-1/PD-L1 inhibitors therapy following RT have emerged as critical factors that may influence toxicity profiles in combination treatment regimens. Although no definitive guidance has been established, current evidence suggests that both the interval between RT completion and immunotherapy initiation, as well as the total duration of immune consolidation therapy, may affect the incidence and severity of treatment-related AEs.

Regarding treatment initiation timing, Data from the PACIFIC^49^^,^^50^^,^^51^ trial recommend initiating durvalumab within 42 days of completing CRT, with the goal of maximizing antitumor immune engagement. Encouragingly, a meta-analysis^108^ of 2,560 patients across nine trials reported a slightly lower incidence of Grade ≥3 pneumonitis in patients initiating durvalumab within this window (4.12%, 95% CI: 2–6%) compared to the overall incidence across all studies (5.36%, 95% CI: 3–8%), with minimal heterogeneity (I^2^ = 0.00%, *p* = 0.56). These data suggest that moderate delays, within 6 weeks post-RT, do not appear to exacerbate pulmonary toxicity and may be well tolerated in most patients. However, other studies present a more nuanced view. For example, in the SABR-5 trial,^96^ delaying systemic therapy until disease progression was associated with a significantly lower risk of Grade ≥2 AEs compared to early initiation around the time of SBRT (OR = 0.35; 95% CI: 0.15–0.70; *p* < 0.001). These observations suggest that both excessively early and overly delayed initiation may influence toxicity, and that the optimal timing window may vary by tumor type and treatment context.

Equally important is the duration of PD-1/PD-L1 inhibitors treatment. The optimal duration of PD-1/PD-L1 inhibitors therapy following RT is also under active investigation. Although 12-month consolidation therapy has been adopted as standard in trials such as PACIFIC, real-world data indicate that many patients discontinue treatment earlier. In a cohort of 1,006 patients with NSCLC, the median duration of durvalumab therapy was 7 months,^109^ with only 31% completing the full 12-month course. Immune-related toxicity was the most common cause of early discontinuation (15%), and the incidence of Grade 3–4 pneumonitis was notably higher than in the PACIFIC trial (10.8% vs. 3.4%). Survival outcomes were influenced by treatment duration. Patients who received 9 months of durvalumab achieved comparable 2-year overall survival to those who completed the full year, whereas those who stopped at 6 months experienced significantly higher mortality (HR = 1.53; 95% CI: 1.25–1.85). These findings suggest that moderate treatment durations may preserve efficacy while reducing cumulative toxicity, but excessively short courses may compromise clinical benefit. Current guidelines generally recommend 1 to 2 years of consolidation immunotherapy, depending on disease stage, response, and tolerance. Extended regimens such as the 2-year GEMSTONE-301 protocol may be appropriate for patients exhibiting partial response or stable disease with favorable treatment tolerance. Conversely, early discontinuation may be justified in patients achieving complete remission or developing Grade ≥3 immune-related AEs, with the option of reinitiating therapy upon relapse.

Overall, the initiation timing and duration of PD-1/PD-L1 inhibitors therapy following RT appear to play a meaningful role in shaping toxicity risk. While moderate delays in therapy (within 6 weeks post-RT) seem tolerable, excessively early or prolonged regimens may increase immune-related adverse events. These results highlight the need to tailor treatment schedules to patient-specific factors such as tumor histology, treatment response, and prior toxicity. Moving forward, prospective, stratified clinical trials comparing different initiation intervals and therapy durations are needed to refine the safe and effective integration of immunotherapy following radiotherapy in various oncologic settings.

### Toxicity considerations in multimodal regimens involving radiotherapy and immunotherapy

The integration of RT with PD-1/PD-L1 Inhibitors has emerged as an important therapeutic option for multiple solid tumors. In recent years, this strategy now incorporates other systemic agents such as chemotherapy, anti-angiogenic therapies, and additional PD-1/PD-L1 inhibitors, thereby offering a broader therapeutic scope. While early clinical data suggest that these multimodal regimens may enhance tumor control and improve survival outcomes, their adoption also raises concerns regarding increased toxicity risks, warranting careful evaluation.

One approach under exploration involves induction chemotherapy and immunotherapy followed by RT. For instance, a retrospective study of 75 patients with unresectable stage III NSCLC reported that induction PD-1/PD-L1 inhibitors combined with chemotherapy, followed by definitive CRT, was associated with acceptable toxicity and a numerically higher two-year overall survival (OS) rate among patients who also received consolidation PD-1/PD-L1 inhibitors.^110^ Similarly, in HCC, retrospective data suggest that Atezolizumab plus Bevacizumab followed by RT may offer a favorable safety profile, with minimal grade ≥2 hepatic events and potential benefits in lymphocyte recovery, which is considered an emerging prognostic factor. These encouraging findings warrant validation in prospective studies.^111^

Another evolving strategy includes RT combined with dual immune checkpoint blockade during consolidation therapy. In the BTCRC LUN16-081 trial, unresectable patients with LA-NSCLC receiving consolidation Nivolumab plus Ipilimumab showed similar median progression-free survival (PFS) and OS compared to Nivolumab alone, with a modest numerical advantage. However, the combination arm experienced a higher frequency of grade ≥3 treatment-related adverse events (TRAEs), suggesting a potential trade-off between enhanced immune activation and increased toxicity.^112^ Additional insights are offered by a multi-trial analysis that assessed SBRT in combination with various systemic immunotherapies. In that study, patients receiving dual ICI regimens (e.g., Nivolumab + Ipilimumab) demonstrated higher rates of grade ≥2 adverse events, including elevations in AST, CPK, and LDH, as well as increased rates of rash and pneumonitis, compared to those on monotherapy.^113^ While these findings highlight possible safety concerns, the absence of matched efficacy data in some cohorts limits definitive conclusions. In HCC, a phase I trial investigating SBRT followed by Durvalumab with or without Ipilimumab also reported improved tumor response and longer PFS and OS in the combination group. However, this was accompanied by a notably higher incidence of grade 3 hepatotoxicity, underscoring the importance of balancing benefit and risk when intensifying immunotherapy.^114^

Collectively, current evidence suggests that multimodal RT-based immunotherapy regimens have the potential to improve oncologic outcomes across tumor types, though they may also contribute to a higher burden of immune-related toxicity, particularly with dual checkpoint blockade. The most frequently observed adverse events include dermatologic reactions, pneumonitis, and hepatic dysfunction, which may impact the treatment continuity and patient quality of life. Given these observations, personalized treatment approaches are essential. Assessing immune status, comorbidities, and tumor-specific features may help optimize patient selection and treatment intensity. Future research efforts should focus on refining combination strategies, identifying predictive biomarkers for toxicity, and establishing individualized thresholds for treatment escalation to maximize clinical benefit while minimizing harm.

## Discussion

Research suggests that PD-1/PD-L1 Inhibitors significantly boost the immune response against tumors in both the irradiated area and throughout the body (local and systemic anti-tumor immunity) when combined with RT. However, this potent synergy may dysregulate the cGAS-STING pathway and cytokine milieu, leading to collateral tissue injury and immune-related toxicities. We provide an overview of safety profiles and potential toxicities of RT+ anti-PD-1/PD-L1 combination therapy. Notably, although an increased incidence of mild (grade 1–2) pneumonitis has been observed in patients with lung cancer, there is currently no consistent evidence of a rise in severe (grade ≥3) pulmonary toxicity. Moreover, combination therapy appears to be well tolerated in the treatment of central nervous system (CNS), head and neck, and hepatic malignancies, supporting its broader clinical applicability. However, patients aged 70 or older may experience a higher incidence of severe (grade 3/4) side effects. This highlights the importance of cautious patient management when treating elderly patients or patients with underlying conditions.

**Figure 3:**
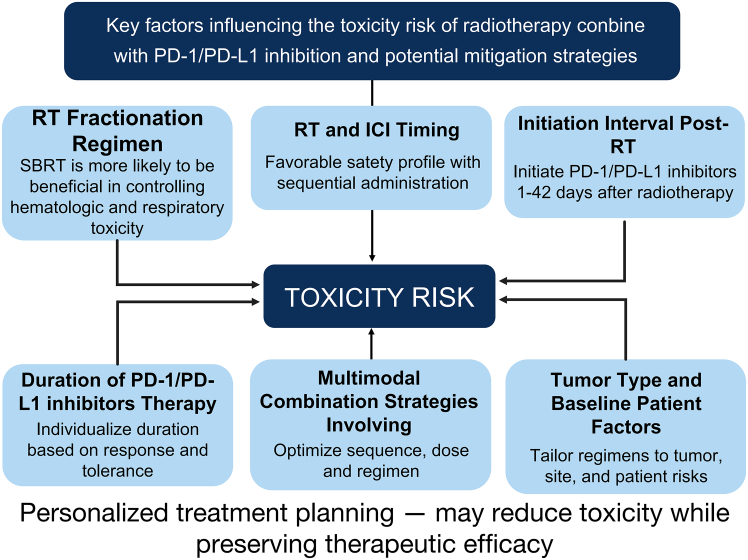
Key determinants and mitigation strategies for toxicity in combined radiotherapy and PD-1/PD-L1 inhibitor therapy Key factors influencing toxicity risk when combining radiotherapy with PD-1/PD-L1 inhibition are illustrated. Factors include RT fractionation regimen, timing of RT and PD-1/PD-L1 inhibitions, interval between RT and PD-1/PD-L1 inhibitions initiation, duration of PD-1/PD-L1 therapy, multimodal combination strategies, and tumor/patient baseline characteristics. Personalized treatment planning based on these factors may help reduce toxicity while maintaining therapeutic efficacy.

Selecting the appropriate radiotherapy fractionation, such as HFRT or SBRT, may help reduce toxicity while enhancing immunogenicity. The optimal timing for initiating PD-1/PD-L1 inhibitors following RT remains an area of active investigation. While early initiation may potentiate immune activation, it may also increase the risk of severe adverse events, such as grade ≥3 pneumonitis. Existing data suggest that initiating PD-1/PD-L1 inhibitors within 42 days of RT, along with prolonged immunotherapy consolidation (≥1 year), may improve safety outcomes, though further prospective validation is needed. Figure 3 summarizes the key determinants of toxicity risk associated with combined radiotherapy and PD-1/PD-L1 Inhibitors therapy, as well as implications for reducing toxicity risk in combination therapy.

Several innovative combination therapy strategies have shown good efficacy, with the strategy of combining chemotherapy with immune induction therapy followed by RT demonstrating favorable efficacy and safety. When RT is combined with dual immunotherapy agents (such as Nivolumab and Ipilimumab), it has been proven to significantly improve PFS and OS. However, this treatment strategy can also significantly increase the incidence of immune-mediated toxicities, such as rash, pneumonia, and liver toxicity. Immunotherapy, by activating the immune system, can potentially lead to an exaggerated immune response, causing damage to normal tissues. Therefore, balancing the potential therapeutic benefits with the risks of AEs becomes crucial. These findings guide clinical practice by optimizing efficacy and minimizing toxicity.

## Limitations of the study

As a comprehensive narrative review, this work has inherent limitations. First, while it systematically synthesizes existing evidence, it can only offer broad inferences regarding toxicity risk and lacks the rigor of formal statistical aggregation or meta-analysis. Second, much of the clinical safety data on combining radiotherapy with PD-1/PD-L1 inhibitors derives from retrospective studies characterized by significant heterogeneity in study design, non-standardized definitions of TRAEs, and inconsistent follow-up durations. These retrospective studies are also inherently vulnerable to selection bias, confounding, and differences in baseline patient characteristics, including disease stage, comorbidities, and prior treatments, all of which may influence both treatment outcomes and toxicity reporting. Third, this review primarily focuses on comparative analyses between combination therapy and monotherapy arms. However, the relative scarcity of well-designed head-to-head trials limits the precision with which toxicity differentials can be assessed.

In the future, conducting rigorously designed prospective trials and adopting standardized toxicity reporting criteria will be critical for validating and expanding these findings. Prospective designs will allow for better control of baseline confounders and enable more accurate attribution of observed adverse events to specific treatment components. Mechanistic studies using translational models, coupled with biomarker-driven strategies, will be essential for predicting AE risk and refining personalized regimens. Ultimately, deepening our understanding of the dynamic interplay between radiation-induced immune modulation and checkpoint inhibition may help optimize therapeutic efficacy while minimizing harm, thereby laying the foundation for the next generation of precision immuno-radiotherapy.

## Acknowledgments

This work was supported by the 10.13039/100014717National Natural Science Foundation of China (No. 82173452), the National Natural Science Foundation of Fujian Province, China (No.2020J011014), and the Excellent Young Scholars Cultivation Project of 10.13039/501100013795Fujian Medical University Union Hospital (2022XH034). During the preparation of this work, the authors used ChatGPT to improve language. After using this tool, the authors reviewed and edited the content as needed and take full responsibility for the content of the publication. The figure is original, and the figure support was created with BioRender.com.

## Author contributions

Z-LJ, Y-J, X-H, L-ZY, and L-Y conducted the study and drafted the article. Z-LJ and Y-J participated in the design and production of the mechanical diagram. Z-R and Y-QW conceived the study and participated in its design and coordination. All the authors read and approved the final article.

## Declaration of interests

The authors declare no competing interests.

## References

[1] Constantinidou A., Alifieris C., Trafalis D.T. (2019). Targeting Programmed Cell Death -1 (PD-1) and Ligand (PD-L1): A new era in cancer active immunotherapy. Pharmacol. Ther..

[2] Apostolidis J., Sayyed A., Darweesh M., Kaloyannidis P., Al Hashmi H. (2020). Current Clinical Applications and Future Perspectives of Immune Checkpoint Inhibitors in Non-Hodgkin Lymphoma. J. Immunol. Res..

[3] Pang K., Shi Z.-D., Wei L.-Y., Dong Y., Ma Y.-Y., Wang W., Wang G.-Y., Cao M.-Y., Dong J.-J., Chen Y.-A. (2023). Research progress of therapeutic effects and drug resistance of immunotherapy based on PD-1/PD-L1 blockade. Drug Resist. Updat..

[4] Li Y., Liu J., Gao L., Liu Y., Meng F., Li X., Qin F.X.-F. (2020). Targeting the tumor microenvironment to overcome immune checkpoint blockade therapy resistance. Immunol. Lett..

[5] Ettinger D.S., Wood D.E., Aisner D.L., Akerley W., Bauman J.R., Bharat A., Bruno D.S., Chang J.Y., Chirieac L.R., DeCamp M. (2023). NCCN Guidelines® Insights: Non-Small Cell Lung Cancer, Version 2.2023. J. Natl. Compr. Canc. Netw..

[6] Swetter S.M., Thompson J.A., Albertini M.R., Barker C.A., Baumgartner J., Boland G., Chmielowski B., DiMaio D., Durham A., Fields R.C. (2021). NCCN Guidelines® Insights: Melanoma: Cutaneous, Version 2.2021. J. Natl. Compr. Canc. Netw..

[7] Benson A.B., D’Angelica M.I., Abrams T., Abbott D.E., Ahmed A., Anaya D.A., Anders R., Are C., Bachini M., Binder D. (2023). NCCN Guidelines® Insights: Biliary Tract Cancers, Version 2.2023. J. Natl. Compr. Canc. Netw..

[8] Deng L., Liang H., Burnette B., Beckett M., Darga T., Weichselbaum R.R., Fu Y.-X. (2014). Irradiation and anti-PD-L1 treatment synergistically promote antitumor immunity in mice. J. Clin. Investig..

[9] Liu S., Kwon M., Mannino M., Yang N., Renda F., Khodjakov A., Pellman D. (2018). Nuclear envelope assembly defects link mitotic errors to chromothripsis. Nature.

[10] Mackenzie K.J., Carroll P., Martin C.-A., Murina O., Fluteau A., Simpson D.J., Olova N., Sutcliffe H., Rainger J.K., Leitch A. (2017). cGAS surveillance of micronuclei links genome instability to innate immunity. Nature.

[11] Durante M., Formenti S.C. (2018). Radiation-Induced Chromosomal Aberrations and Immunotherapy: Micronuclei, Cytosolic DNA, and Interferon-Production Pathway. Front. Oncol..

[12] Skopelja-Gardner S., An J., Elkon K.B. (2022). Role of the cGAS-STING pathway in systemic and organ-specific diseases. Nat. Rev. Nephrol..

[13] Sun L., Wu J., Du F., Chen X., Chen Z.J. (2013). Cyclic GMP-AMP synthase is a cytosolic DNA sensor that activates the type I interferon pathway. Science.

[14] Ishikawa H., Ma Z., Barber G.N. (2009). STING regulates intracellular DNA-mediated, type I interferon-dependent innate immunity. Nature.

[15] Dong M., Fitzgerald K.A. (2024). DNA-sensing pathways in health, autoinflammatory and autoimmune diseases. Nat. Immunol..

[16] Tubbs A., Nussenzweig A. (2017). Endogenous DNA Damage as a Source of Genomic Instability in Cancer. Cell.

[17] Du S.-S., Chen G.-W., Yang P., Chen Y.-X., Hu Y., Zhao Q.-Q., Zhang Y., Liu R., Zheng D.-X., Zhou J. (2022). Radiation Therapy Promotes Hepatocellular Carcinoma Immune Cloaking via PD-L1 Upregulation Induced by cGAS-STING Activation. Int. J. Radiat. Oncol. Biol. Phys..

[18] Mouw K.W., Goldberg M.S., Konstantinopoulos P.A., D’Andrea A.D. (2017). DNA Damage and Repair Biomarkers of Immunotherapy Response. Cancer Discov..

[19] Chen D.S., Mellman I. (2013). Oncology meets immunology: the cancer-immunity cycle. Immunity.

[20] Apetoh L., Ghiringhelli F., Tesniere A., Obeid M., Ortiz C., Criollo A., Mignot G., Maiuri M.C., Ullrich E., Saulnier P. (2007). Toll-like receptor 4-dependent contribution of the immune system to anticancer chemotherapy and radiotherapy. Nat. Med..

[21] Obeid M., Tesniere A., Ghiringhelli F., Fimia G.M., Apetoh L., Perfettini J.-L., Castedo M., Mignot G., Panaretakis T., Casares N. (2007). Calreticulin exposure dictates the immunogenicity of cancer cell death. Nat. Med..

[22] Ghiringhelli F., Apetoh L., Tesniere A., Aymeric L., Ma Y., Ortiz C., Vermaelen K., Panaretakis T., Mignot G., Ullrich E. (2009). Activation of the NLRP3 inflammasome in dendritic cells induces IL-1beta-dependent adaptive immunity against tumors. Nat. Med..

[23] Kabiljo J., Harpain F., Carotta S., Bergmann M. (2019). Radiotherapy as a Backbone for Novel Concepts in Cancer Immunotherapy. Cancers (Basel).

[24] Wu Y., Yi M., Niu M., Zhou B., Mei Q., Wu K. (2024). Beyond success: unveiling the hidden potential of radiotherapy and immunotherapy in solid tumors. Cancer Commun..

[25] Shen M., Jiang X., Peng Q., Oyang L., Ren Z., Wang J., Peng M., Zhou Y., Deng X., Liao Q. (2025). The cGAS‒STING pathway in cancer immunity: mechanisms, challenges, and therapeutic implications. J. Hematol. Oncol..

[26] Liu Z., Wang D., Zhang J., Xiang P., Zeng Z., Xiong W., Shi L. (2023). cGAS-STING signaling in the tumor microenvironment. Cancer Lett..

[27] Zheng J., Mo J., Zhu T., Zhuo W., Yi Y., Hu S., Yin J., Zhang W., Zhou H., Liu Z. (2020). Comprehensive elaboration of the cGAS-STING signaling axis in cancer development and immunotherapy. Mol. Cancer.

[28] Hsu P., Santner-Nanan B., Hu M., Skarratt K., Lee C.H., Stormon M., Wong M., Fuller S.J., Nanan R. (2015). IL-10 Potentiates Differentiation of Human Induced Regulatory T Cells via STAT3 and Foxo1. J. Immunol..

[29] Beauford S.S., Kumari A., Garnett-Benson C. (2020). Ionizing radiation modulates the phenotype and function of human CD4+ induced regulatory T cells. BMC Immunol..

[30] Wang Q., Bergholz J.S., Ding L., Lin Z., Kabraji S.K., Hughes M.E., He X., Xie S., Jiang T., Wang W. (2022). STING agonism reprograms tumor-associated macrophages and overcomes resistance to PARP inhibition in BRCA1-deficient models of breast cancer. Nat. Commun..

[31] Liu X., Hogg G.D., Zuo C., Borcherding N.C., Baer J.M., Lander V.E., Kang L.-I., Knolhoff B.L., Ahmad F., Osterhout R.E. (2023). Context-dependent activation of STING-interferon signaling by CD11b agonists enhances anti-tumor immunity. Cancer Cell.

[32] Li C., Jiang P., Wei S., Xu X., Wang J. (2020). Regulatory T cells in tumor microenvironment: new mechanisms, potential therapeutic strategies and future prospects. Mol. Cancer.

[33] Prakash H., Klug F., Nadella V., Mazumdar V., Schmitz-Winnenthal H., Umansky L. (2016). Low doses of gamma irradiation potentially modifies immunosuppressive tumor microenvironment by retuning tumor-associated macrophages: lesson from insulinoma. Carcinogenesis.

[34] Li M., Gan L., Song A., Xue J., Lu Y. (2019). Rethinking pulmonary toxicity in advanced non-small cell lung cancer in the era of combining anti-PD-1/PD-L1 therapy with thoracic radiotherapy. Biochim. Biophys. Acta. Rev. Cancer.

[35] Bangert A., Andrassy M., Müller A.-M., Bockstahler M., Fischer A., Volz C.H., Leib C., Göser S., Korkmaz-Icöz S., Zittrich S. (2016). Critical role of RAGE and HMGB1 in inflammatory heart disease. Proc. Natl. Acad. Sci. USA.

[36] Myers C.J., Lu B. (2017). Decreased Survival After Combining Thoracic Irradiation and an Anti-PD-1 Antibody Correlated With Increased T-cell Infiltration Into Cardiac and Lung Tissues. Int. J. Radiat. Oncol. Biol. Phys..

[37] Johnson D.B., Balko J.M., Compton M.L., Chalkias S., Gorham J., Xu Y., Hicks M., Puzanov I., Alexander M.R., Bloomer T.L. (2016). Fulminant Myocarditis with Combination Immune Checkpoint Blockade. N. Engl. J. Med..

[38] Campbell I.L., Krucker T., Steffensen S., Akwa Y., Powell H.C., Lane T., Carr D.J., Gold L.H., Henriksen S.J., Siggins G.R. (1999). Structural and functional neuropathology in transgenic mice with CNS expression of IFN-alpha. Brain Res..

[39] Ormandy L.A., Hillemann T., Wedemeyer H., Manns M.P., Greten T.F., Korangy F. (2005). Increased populations of regulatory T cells in peripheral blood of patients with hepatocellular carcinoma. Cancer Res..

[40] Gao Q., Qiu S.-J., Fan J., Zhou J., Wang X.-Y., Xiao Y.-S., Xu Y., Li Y.-W., Tang Z.-Y. (2007). Intratumoral balance of regulatory and cytotoxic T cells is associated with prognosis of hepatocellular carcinoma after resection. J. Clin. Oncol..

[41] Shao L., Luo Y., Zhou D. (2014). Hematopoietic stem cell injury induced by ionizing radiation. Antioxid. Redox Signal..

[42] Esfahani K., Elkrief A., Calabrese C., Lapointe R., Hudson M., Routy B., Miller W.H., Calabrese L. (2020). Moving towards personalized treatments of immune-related adverse events. Nat. Rev. Clin. Oncol..

[43] Wang Y., Wiesnoski D.H., Helmink B.A., Gopalakrishnan V., Choi K., DuPont H.L., Jiang Z.-D., Abu-Sbeih H., Sanchez C.A., Chang C.-C. (2018). Fecal microbiota transplantation for refractory immune checkpoint inhibitor-associated colitis. Nat. Med..

[44] Routy B., Le Chatelier E., Derosa L., Duong C.P.M., Alou M.T., Daillère R., Fluckiger A., Messaoudene M., Rauber C., Roberti M.P. (2018). Gut microbiome influences efficacy of PD-1-based immunotherapy against epithelial tumors. Science.

[45] Hepkema J., Lee N.K., Stewart B.J., Ruangroengkulrith S., Charoensawan V., Clatworthy M.R., Hemberg M. (2023). Predicting the impact of sequence motifs on gene regulation using single-cell data. Genome Biol..

[46] Axelrod M.L., Meijers W.C., Screever E.M., Qin J., Carroll M.G., Sun X., Tannous E., Zhang Y., Sugiura A., Taylor B.C. (2022). T cells specific for α-myosin drive immunotherapy-related myocarditis. Nature.

[47] Shaverdian N., Lisberg A.E., Bornazyan K., Veruttipong D., Goldman J.W., Formenti S.C., Garon E.B., Lee P. (2017). Previous radiotherapy and the clinical activity and toxicity of pembrolizumab in the treatment of non-small-cell lung cancer: a secondary analysis of the KEYNOTE-001 phase 1 trial. Lancet Oncol..

[48] Edwards D.M., Sankar K., Alseri A., Jiang R., Schipper M., Miller S., Dess K., Strohbehn G.W., Elliott D.A., Moghanaki D. (2024). Pneumonitis After Chemoradiotherapy and Adjuvant Durvalumab in Stage III Non-Small Cell Lung Cancer. Int. J. Radiat. Oncol. Biol. Phys..

[49] Antonia S.J., Villegas A., Daniel D., Vicente D., Murakami S., Hui R., Kurata T., Chiappori A., Lee K.H., de Wit M. (2018). Overall Survival with Durvalumab after Chemoradiotherapy in Stage III NSCLC. N. Engl. J. Med..

[50] Gray J.E., Villegas A., Daniel D., Vicente D., Murakami S., Hui R., Kurata T., Chiappori A., Lee K.H., Cho B.C. (2020). Three-Year Overall Survival with Durvalumab after Chemoradiotherapy in Stage III NSCLC-Update from PACIFIC. J. Thorac. Oncol..

[51] Antonia S.J., Villegas A., Daniel D., Vicente D., Murakami S., Hui R., Yokoi T., Chiappori A., Lee K.H., de Wit M. (2017). Durvalumab after Chemoradiotherapy in Stage III Non-Small-Cell Lung Cancer. N. Engl. J. Med..

[52] Geng Y., Zhang Q., Feng S., Li C., Wang L., Zhao X., Yang Z., Li Z., Luo H., Liu R. (2021). Safety and Efficacy of PD-1/PD-L1 inhibitors combined with radiotherapy in patients with non-small-cell lung cancer: a systematic review and meta-analysis. Cancer Med..

[53] Socinski M.A., Özgüroğlu M., Villegas A., Daniel D., Vicente D., Murakami S., Hui R., Gray J.E., Park K., Vincent M. (2021). Durvalumab After Concurrent Chemoradiotherapy in Elderly Patients With Unresectable Stage III Non-Small-Cell Lung Cancer (PACIFIC). Clin. Lung Cancer.

[54] Fiorica F., Belluomini L., Stefanelli A., Santini A., Urbini B., Giorgi C., Frassoldati A. (2018). Immune Checkpoint Inhibitor Nivolumab and Radiotherapy in Pretreated Lung Cancer Patients: Efficacy and Safety of Combination. Am. J. Clin. Oncol..

[55] Ratnayake G., Shanker M., Roberts K., Mason R., Hughes B.G.M., Lwin Z., Jain V., O’Byrne K., Lehman M., Chua B. (2020). Prior or concurrent radiotherapy and nivolumab immunotherapy in non-small cell lung cancer. Asia Pac. J. Clin. Oncol..

[56] Yamaguchi O., Kaira K., Hashimoto K., Mouri A., Miura Y., Shiono A., Nishihara F., Murayama Y., Noda S.E., Kato S. (2019). Radiotherapy is an independent prognostic marker of favorable prognosis in non-small cell lung cancer patients after treatment with the immune checkpoint inhibitor, nivolumab. Thorac. Cancer.

[57] Theelen W.S.M.E., Peulen H.M.U., Lalezari F., van der Noort V., de Vries J.F., Aerts J.G.J.V., Dumoulin D.W., Bahce I., Niemeijer A.-L.N., de Langen A.J. (2019). Effect of Pembrolizumab After Stereotactic Body Radiotherapy vs Pembrolizumab Alone on Tumor Response in Patients With Advanced Non-Small Cell Lung Cancer: Results of the PEMBRO-RT Phase 2 Randomized Clinical Trial. JAMA Oncol..

[58] Welsh J., Menon H., Chen D., Verma V., Tang C., Altan M., Hess K., de Groot P., Nguyen Q.-N., Varghese R. (2020). Pembrolizumab with or without radiation therapy for metastatic non-small cell lung cancer: a randomized phase I/II trial. J. Immunother. Cancer.

[59] Pakkala S., Higgins K., Chen Z., Sica G., Steuer C., Zhang C., Zhang G., Wang S., Hossain M.S., Nazha B. (2020). Durvalumab and tremelimumab with or without stereotactic body radiation therapy in relapsed small cell lung cancer: a randomized phase II study. J. Immunother. Cancer.

[60] Samuel E., Lie G., Balasubramanian A., Hiong A., So Y., Voskoboynik M., Moore M., Shackleton M., Haydon A., John T. (2021). Impact of Radiotherapy on the Efficacy and Toxicity of anti-PD-1 Inhibitors in Metastatic NSCLC. Clin. Lung Cancer.

[61] Wang P., Yin T., Zhao K., Yu J., Teng F. (2022). Efficacy of single-site radiotherapy plus PD-1 inhibitors vs PD-1 inhibitors for oligometastatic non-small cell lung cancer. J. Cancer Res. Clin. Oncol..

[62] Hosokawa S., Ichihara E., Bessho A., Harada D., Inoue K., Shibayama T., Kishino D., Harita S., Ochi N., Oda N. (2021). Erratum to: Impact of previous thoracic radiation therapy on the efficacy of immune checkpoint inhibitors in advanced non-small-cell lung cancer. Jpn. J. Clin. Oncol..

[63] Saito G., Oya Y., Taniguchi Y., Kawachi H., Daichi F., Matsumoto H., Iwasawa S., Suzuki H., Niitsu T., Miyauchi E. (2021). Real-world survey of pneumonitis and its impact on durvalumab consolidation therapy in patients with non-small cell lung cancer who received chemoradiotherapy after durvalumab approval (HOPE-005/CRIMSON). Lung Cancer.

[64] Altorki N.K., McGraw T.E., Borczuk A.C., Saxena A., Port J.L., Stiles B.M., Lee B.E., Sanfilippo N.J., Scheff R.J., Pua B.B. (2021). Neoadjuvant durvalumab with or without stereotactic body radiotherapy in patients with early-stage non-small-cell lung cancer: a single-centre, randomised phase 2 trial. Lancet Oncol..

[65] Zhou Q., Chen M., Jiang O., Pan Y., Hu D., Lin Q., Wu G., Cui J., Chang J., Cheng Y. (2022). Sugemalimab versus placebo after concurrent or sequential chemoradiotherapy in patients with locally advanced, unresectable, stage III non-small-cell lung cancer in China (GEMSTONE-301): interim results of a randomised, double-blind, multicentre, phase 3 trial. Lancet Oncol..

[66] Cagney D.N., Martin A.M., Catalano P.J., Redig A.J., Lin N.U., Lee E.Q., Wen P.Y., Dunn I.F., Bi W.L., Weiss S.E. (2017). Incidence and prognosis of patients with brain metastases at diagnosis of systemic malignancy: a population-based study. Neuro Oncol..

[67] Vogelbaum M.A., Brown P.D., Messersmith H., Brastianos P.K., Burri S., Cahill D., Dunn I.F., Gaspar L.E., Gatson N.T.N., Gondi V. (2022). Treatment for Brain Metastases: ASCO-SNO-ASTRO Guideline. J. Clin. Oncol..

[68] Hubbeling H.G., Schapira E.F., Horick N.K., Goodwin K.E.H., Lin J.J., Oh K.S., Shaw A.T., Mehan W.A., Shih H.A., Gainor J.F. (2018). Safety of Combined PD-1 Pathway Inhibition and Intracranial Radiation Therapy in Non-Small Cell Lung Cancer. J. Thorac. Oncol..

[69] Shepard M.J., Xu Z., Donahue J., Eluvathingal Muttikkal T.J., Cordeiro D., Hansen L., Mohammed N., Gentzler R.D., Larner J., Fadul C.E., Sheehan J.P. (2020). Stereotactic radiosurgery with and without checkpoint inhibition for patients with metastatic non-small cell lung cancer to the brain: a matched cohort study. J. Neurosurg..

[70] Scoccianti S., Olmetto E., Pinzi V., Osti M.F., Di Franco R., Caini S., Anselmo P., Matteucci P., Franceschini D., Mantovani C. (2021). Immunotherapy in association with stereotactic radiotherapy for non-small cell lung cancer brain metastases: results from a multicentric retrospective study on behalf of AIRO. Neuro Oncol..

[71] Abdulhaleem M., Johnston H., D’Agostino R., Lanier C., LeCompte M., Cramer C.K., Ruiz J., Lycan T., Lo H.-W., Watabe K. (2022). Local control outcomes for combination of stereotactic radiosurgery and immunotherapy for non-small cell lung cancer brain metastases. J. Neuro Oncol..

[72] Li J., Li W., Xu S., Zhu H. (2023). Brain injury after cranial radiotherapy combined with immunotherapy for brain metastases in lung cancer: a retrospective study. Future Oncol..

[73] Ma J., Tian Y., Hao S., Zheng L., Hu W., Zhai X., Meng D., Zhu H. (2022). Outcomes of first-line anti-PD-L1 blockades combined with brain radiotherapy for extensive-stage small-cell lung cancer with brain metastasis. J. Neuro Oncol..

[74] Singh C., Qian J.M., Yu J.B., Chiang V.L. (2020). Local tumor response and survival outcomes after combined stereotactic radiosurgery and immunotherapy in non-small cell lung cancer with brain metastases. J. Neurosurg..

[75] Theelen W.S.M.E., Chen D., Verma V., Hobbs B.P., Peulen H.M.U., Aerts J.G.J.V., Bahce I., Niemeijer A.L.N., Chang J.Y., de Groot P.M. (2021). Pembrolizumab with or without radiotherapy for metastatic non-small-cell lung cancer: a pooled analysis of two randomised trials. Lancet Respir. Med..

[76] Lu S., Guo X., Yang Z., Sun Y., Niu J., Jing X., Zhu H. (2024). Immunotherapy combined with cranial radiotherapy for driver-negative non-small-cell lung cancer brain metastases: a retrospective study. Future Oncol..

[77] Guo T., Zhou Y., Liang F., Wang Z., Bourbonne V., Käsmann L., Sundahl N., Wu A.J.-C., Ni J., Zhu Z. (2024). Potential synergistic effects of cranial radiotherapy and atezolizumab in non-small cell lung cancer: an analysis of individual patient data from seven prospective trials. Transl. Lung Cancer Res..

[78] Guo Y., Luo Y., Zhang Q., Huang X., Li Z., Shen L., Feng J., Sun Y., Yang K., Ge M. (2021). First-line treatment with chemotherapy plus cetuximab in Chinese patients with recurrent and/or metastatic squamous cell carcinoma of the head and neck: Efficacy and safety results of the randomised, phase III CHANGE-2 trial. Eur. J. Cancer.

[79] Su Z.Y., Siak P.Y., Lwin Y.Y., Cheah S.-C. (2024). Epidemiology of nasopharyngeal carcinoma: current insights and future outlook. Cancer Metastasis Rev..

[80] Tao Y., Aupérin A., Sun X., Sire C., Martin L., Coutte A., Lafond C., Miroir J., Liem X., Rolland F. (2020). Avelumab-cetuximab-radiotherapy versus standards of care in locally advanced squamous-cell carcinoma of the head and neck: The safety phase of a randomised phase III trial GORTEC 2017-01 (REACH). Eur. J. Cancer.

[81] Tao Y., Biau J., Sun X.S., Sire C., Martin L., Alfonsi M., Prevost J.B., Modesto A., Lafond C., Tourani J.M. (2023). Pembrolizumab versus cetuximab concurrent with radiotherapy in patients with locally advanced squamous cell carcinoma of head and neck unfit for cisplatin (GORTEC 2015-01 PembroRad): a multicenter, randomized, phase II trial. Ann. Oncol..

[82] Liu X., Zhang Y., Yang K.-Y., Zhang N., Jin F., Zou G.-R., Zhu X.-D., Xie F.-Y., Liang X.-Y., Li W.-F. (2024). Induction-concurrent chemoradiotherapy with or without sintilimab in patients with locoregionally advanced nasopharyngeal carcinoma in China (CONTINUUM): a multicentre, open-label, parallel-group, randomised, controlled, phase 3 trial. Lancet.

[83] Sung H., Ferlay J., Siegel R.L., Laversanne M., Soerjomataram I., Jemal A., Bray F. (2021). Global Cancer Statistics 2020: GLOBOCAN Estimates of Incidence and Mortality Worldwide for 36 Cancers in 185 Countries. CA Cancer J. Clin..

[84] Su K., Guo L., Ma W., Wang J., Xie Y., Rao M., Zhang J., Li X., Wen L., Li B. (2022). PD-1 inhibitors plus anti-angiogenic therapy with or without intensity-modulated radiotherapy for advanced hepatocellular carcinoma: A propensity score matching study. Front. Immunol..

[85] Zhang R.-J., Zhou H.-M., Lu H.-Y., Yu H.-P., Tang W.-Z., Qiu M.-Q., Yan L.-Y., Long M.-Y., Su T.-S., Xiang B.-D. (2023). Radiotherapy plus anti-PD1 versus radiotherapy for hepatic toxicity in patients with hepatocellular carcinoma. Radiat. Oncol..

[86] Li J.-X., Su T.-S., Gong W.-F., Zhong J.-H., Yan L.-Y., Zhang J., Li L.-Q., He M.-L., Zhang R.-J., Du Y.-Q. (2022). Combining stereotactic body radiotherapy with camrelizumab for unresectable hepatocellular carcinoma: a single-arm trial. Hepatol. Int..

[87] Qin S., Ren Z., Meng Z., Chen Z., Chai X., Xiong J., Bai Y., Yang L., Zhu H., Fang W. (2020). Camrelizumab in patients with previously treated advanced hepatocellular carcinoma: a multicentre, open-label, parallel-group, randomised, phase 2 trial. Lancet Oncol..

[88] Bujold A., Massey C.A., Kim J.J., Brierley J., Cho C., Wong R.K.S., Dinniwell R.E., Kassam Z., Ringash J., Cummings B. (2013). Sequential phase I and II trials of stereotactic body radiotherapy for locally advanced hepatocellular carcinoma. J. Clin. Oncol..

[89] Gkika E., Schultheiss M., Bettinger D., Maruschke L., Neeff H.P., Schulenburg M., Adebahr S., Kirste S., Nestle U., Thimme R. (2017). Excellent local control and tolerance profile after stereotactic body radiotherapy of advanced hepatocellular carcinoma. Radiat. Oncol..

[90] Lo S.S., Fakiris A.J., Chang E.L., Mayr N.A., Wang J.Z., Papiez L., Teh B.S., McGarry R.C., Cardenes H.R., Timmerman R.D. (2010). Stereotactic body radiation therapy: a novel treatment modality. Nat. Rev. Clin. Oncol..

[91] Schoenfeld J.D., Giobbie-Hurder A., Ranasinghe S., Kao K.Z., Lako A., Tsuji J., Liu Y., Brennick R.C., Gentzler R.D., Lee C. (2022). Durvalumab plus tremelimumab alone or in combination with low-dose or hypofractionated radiotherapy in metastatic non-small-cell lung cancer refractory to previous PD(L)-1 therapy: an open-label, multicentre, randomised, phase 2 trial. Lancet Oncol..

[92] Wild A.T., Herman J.M., Dholakia A.S., Moningi S., Lu Y., Rosati L.M., Hacker-Prietz A., Assadi R.K., Saeed A.M., Pawlik T.M. (2016). Lymphocyte-Sparing Effect of Stereotactic Body Radiation Therapy in Patients With Unresectable Pancreatic Cancer. Int. J. Radiat. Oncol. Biol. Phys..

[93] Tang C., Liao Z., Gomez D., Levy L., Zhuang Y., Gebremichael R.A., Hong D.S., Komaki R., Welsh J.W. (2014). Lymphopenia association with gross tumor volume and lung V5 and its effects on non-small cell lung cancer patient outcomes. Int. J. Radiat. Oncol. Biol. Phys..

[94] Rodriguez-Ruiz M.E., Buqué A., Hensler M., Chen J., Bloy N., Petroni G., Sato A., Yamazaki T., Fucikova J., Galluzzi L. (2019). Apoptotic caspases inhibit abscopal responses to radiation and identify a new prognostic biomarker for breast cancer patients. OncoImmunology.

[95] Marchi S., Guilbaud E., Tait S.W.G., Yamazaki T., Galluzzi L. (2023). Mitochondrial control of inflammation. Nat. Rev. Immunol..

[96] Prasanna A., Ahmed M.M., Mohiuddin M., Coleman C.N. (2014). Exploiting sensitization windows of opportunity in hyper and hypo-fractionated radiation therapy. J. Thorac. Dis..

[97] Pujol J.-L. (2022). Durvalumab Induces Sustained Survival Benefit After Concurrent Chemoradiotherapy in Stage III Non-Small-Cell Lung Cancer. J. Clin. Oncol..

[98] Baker S., Lechner L., Liu M., Chang J.S., Cruz-Lim E.M., Mou B., Jiang W., Bergman A., Schellenberg D., Alexander A. (2024). Upfront Versus Delayed Systemic Therapy in Patients With Oligometastatic Cancer Treated With SABR in the Phase 2 SABR-5 Trial. Int. J. Radiat. Oncol. Biol. Phys..

[99] Fang P., Jiang W., Allen P., Glitza I., Guha N., Hwu P., Ghia A., Phan J., Mahajan A., Tawbi H., Li J. (2017). Radiation necrosis with stereotactic radiosurgery combined with CTLA-4 blockade and PD-1 inhibition for treatment of intracranial disease in metastatic melanoma. J. Neuro Oncol..

[100] Li J., Wang Y., Tang C., Welsh J.W., Guha-Thakurta N., Carter B.W., Wefel J.S., Ghia A.J., Yeboa D.N., McAleer M.F. (2020). Concurrent Nivolumab And Ipilimumab With Brain Stereotactic Radiosurgery For Brain Metastases From Non-Small Cell Lung Cancer: A Phase I Trial. Int. J. Radiat. Oncol. Biol. Phys..

[101] An Y., Jiang W., Kim B.Y.S., Qian J.M., Tang C., Fang P., Logan J., D’Souza N.M., Haydu L.E., Wang X.A. (2017). Stereotactic radiosurgery of early melanoma brain metastases after initiation of anti-CTLA-4 treatment is associated with improved intracranial control. Radiother. Oncol..

[102] Schapira E., Hubbeling H., Yeap B.Y., Mehan W.A., Shaw A.T., Oh K., Gainor J.F., Shih H.A. (2018). Improved Overall Survival and Locoregional Disease Control With Concurrent PD-1 Pathway Inhibitors and Stereotactic Radiosurgery for Lung Cancer Patients With Brain Metastases. Int. J. Radiat. Oncol. Biol. Phys..

[103] Porte J., Saint-Martin C., Frederic-Moreau T., Massiani M.-A., Bozec L., Cao K., Verrelle P., Otz J., Jadaud E., Minsat M. (2022). Efficacy and Safety of Combined Brain Stereotactic Radiotherapy and Immune Checkpoint Inhibitors in Non-Small-Cell Lung Cancer with Brain Metastases. Biomedicines.

[104] Trommer M., Adams A., Celik E., Fan J., Funken D., Herter J.M., Linde P., Morgenthaler J., Wegen S., Mauch C. (2022). Oncologic Outcome and Immune Responses of Radiotherapy with Anti-PD-1 Treatment for Brain Metastases Regarding Timing and Benefiting Subgroups. Cancers (Basel).

[105] Chen L., Douglass J., Kleinberg L., Ye X., Marciscano A.E., Forde P.M., Brahmer J., Lipson E., Sharfman W., Hammers H. (2018). Concurrent Immune Checkpoint Inhibitors and Stereotactic Radiosurgery for Brain Metastases in Non-Small Cell Lung Cancer, Melanoma, and Renal Cell Carcinoma. Int. J. Radiat. Oncol. Biol. Phys..

[106] Imber B.S., Hellmann M.D., Kris M.G., Santomasso B.D., Callahan M.K., Osorio J., Rizvi H., Chan T.A., Yang T.J., Yamada Y., Beal K. (2017). Lesion Response and Intracranial Control of Brain Metastases From Non–small Cell Lung Cancer After Stereotactic Radiosurgery or Hypofractionated Radiation Therapy Combined With Checkpoint Inhibitors. Int. J. Radiat. Oncol. Biol. Phys..

[107] Gunderson A.J., Young K.H. (2018). Exploring optimal sequencing of radiation and immunotherapy combinations. Adv. Radiat. Oncol..

[108] Yang Z., Zhong W., Luo Y., Wu C. (2023). The timing of durvalumab administration affects the risk of pneumonitis in patients with locally advanced non-small cell lung cancer: a systematic review and meta-analysis. BMC Cancer.

[109] Bryant A.K., Sankar K., Zhao L., Strohbehn G.W., Elliott D., Moghanaki D., Kelley M.J., Ramnath N., Green M.D. (2022). De-escalating adjuvant durvalumab treatment duration in stage III non-small cell lung cancer. Eur. J. Cancer.

[110] Wang Y., Zhang T., Wang J., Zhou Z., Liu W., Xiao Z., Deng L., Feng Q., Wang X., Lv J. (2023). Induction Immune Checkpoint Inhibitors and Chemotherapy Before Definitive Chemoradiation Therapy for Patients With Bulky Unresectable Stage III Non-Small Cell Lung Cancer. Int. J. Radiat. Oncol. Biol. Phys..

[111] Manzar G.S., De B.S., Abana C.O., Lee S.S., Javle M., Kaseb A.O., Vauthey J.-N., Tran Cao H.S., Koong A.C., Smith G.L. (2022). Outcomes and Toxicities of Modern Combined Modality Therapy with Atezolizumab Plus Bevacizumab and Radiation Therapy for Hepatocellular Carcinoma. Cancers (Basel).

[112] Durm G.A., Mamdani H., Althouse S.K., Jabbour S.K., Ganti A.K., Jalal S.I., Chesney J.A., Naidoo J., Hrinczenko B., Fidler M.J.J. (2022). Consolidation nivolumab plus ipilimumab or nivolumab alone following concurrent chemoradiation for patients with unresectable stage III non-small cell lung cancer: BTCRC LUN 16-081. J. Clin. Orthod..

[113] Lynch C., Korpics M.C., Katipally R.R., Bestvina C.M., Pitroda S.P., Patel J.D., Luke J.J., Chmura S.J., Juloori A. (2024). Safety of combined ablative radiotherapy and immune checkpoint inhibitors in three phase I trials. Eur. J. Cancer.

[114] Juloori A., Katipally R.R.,, Lemons J.M., Singh A.K., Iyer R., Robbins J.R., George B., Hall W.A., Pitroda S.P., Arif F. (2023). Phase 1 Randomized Trial of Stereotactic Body Radiation Therapy Followed by Nivolumab plus Ipilimumab or Nivolumab Alone in Advanced/Unresectable Hepatocellular Carcinoma. Int. J. Radiat. Oncol. Biol. Phys..

